# Analysis of X-ray Structures of Matrix Metalloproteinases via Chaotic Map Clustering

**DOI:** 10.1186/1471-2105-11-500

**Published:** 2010-10-08

**Authors:** Ilenia Giangreco, Orazio Nicolotti, Angelo Carotti, Francesco De Carlo, Gianfranco Gargano, Roberto Bellotti

**Affiliations:** 1 Dipartimento Farmaco-Chimico, University of Bari, via Orabona 4, 70125 Bari, Italy; 2 Dipartimento Interateneo di Fisica, University of Bari, via Amendola 173, 70125 Bari, Italy

## Abstract

**Background:**

Matrix metalloproteinases (MMPs) are well-known biological targets implicated in tumour progression, homeostatic regulation, innate immunity, impaired delivery of pro-apoptotic ligands, and the release and cleavage of cell-surface receptors. With this in mind, the perception of the intimate relationships among diverse MMPs could be a solid basis for accelerated learning in designing new selective MMP inhibitors. In this regard, decrypting the latent molecular reasons in order to elucidate similarity among MMPs is a key challenge.

**Results:**

We describe a pairwise variant of the non-parametric chaotic map clustering (CMC) algorithm and its application to 104 X-ray MMP structures. In this analysis electrostatic potentials are computed and used as input for the CMC algorithm. It was shown that differences between proteins reflect genuine variation of their electrostatic potentials. In addition, the analysis has been also extended to analyze the protein primary structures and the molecular shapes of the MMP co-crystallised ligands.

**Conclusions:**

The CMC algorithm was shown to be a valuable tool in knowledge acquisition and transfer from MMP structures. Based on the variation of electrostatic potentials, CMC was successful in analysing the MMP target family landscape and different subsites. The first investigation resulted in rational figure interpretation of both domain organization as well as of substrate specificity classifications. The second made it possible to distinguish the MMP classes, demonstrating the high specificity of the S_1_' pocket, to detect both the occurrence of punctual mutations of ionisable residues and different side-chain conformations that likely account for induced-fit phenomena. In addition, CMC demonstrated a potential comparable to the most popular UPGMA (Unweighted Pair Group Method with Arithmetic mean) method that, at present, represents a standard clustering bioinformatics approach. Interestingly, CMC and UPGMA resulted in closely comparable outcomes, but often CMC produced more informative and more easy interpretable dendrograms. Finally, CMC was successful for standard pairwise analysis (i.e., Smith-Waterman algorithm) of protein sequences and was used to convincingly explain the complementarity existing between the molecular shapes of the co-crystallised ligand molecules and the accessible MMP void volumes.

## Background

Matrix metalloproteinases (MMPs) are members of the large family of zinc-containing endopeptidases and are biologically attractive drug targets owing to their involvement in tissue remodelling and degradation of extracellular matrix [1]. Allegedly, interest in MMPs was recently prompted by evidence that a number of synthetic inhibitors used for the treatment of various pathological states, such as inflammation, arthritis, and cancer, triggered unbalanced and, to some extent, unexpected responses of certain MMPs; in this respect, MMPs have been distinguished as targets, anti-targets and counter-targets [2]. MMP catalytic domains possess high sequence similarity (56-64%) with a common residue motif, HExGHxxGxxH, incorporating 3 histidines that coordinate the catalytic zinc ion. All protein structures exhibit the characteristic fold of zinc-dependent endopeptidases consisting of a five-stranded beta sheet (1 anti-parallel and 4 parallel) and three alpha helices. Shaped as a cavity crossing the entire enzyme, the active site is characterized by a number of subsites [3] directly involved in the interaction with physiological substrates and natural or synthetic inhibitors. The human genome sequence has enabled us to characterize the entire MMP family, a gallery of proteases encoded by 26 distinct genes. This family includes the archetypal MMPs, the matrylisin, the gelatinases and the convertase-activable MMPs [4]. To date, at least 26 human MMPs are known and diverse efforts for their classification have been made. In view of this, the development of new analytical strategies enabling the decoding and proper interpretation of information encrypted in protein structures is indeed an open challenge. Among others, cluster analysis is a valuable approach to this end. Clustering deals with the partitioning of a set of N elements into K groups based on a suitable similarity criterion. As is well known, clustering is generally performed through parametric and non-parametric methods [5]. The parametric algorithms require prior knowledge of the data structure, enabling the formulation of assumptions, such as establishing the number of clusters to be found. The clustering problem is, thus, converted into an optimization task, as a cost function is minimized in correspondence to the best partition of the data: typical examples are K-means and deterministic annealing. Non-parametric methods represent the optimal strategy when no prior knowledge of potential clusters is available: these methods make few assumptions about the structure of the data. Examples of non-parametric methods are linkage (agglomerative and divisive) algorithms, whose output is a dendrogram displaying the complete hierarchy of clustering solutions on different scales. A recently proposed non-parametric method is chaotic map clustering (CMC) [6]. This algorithm was inspired by a study of the statistical properties of chaotic physical systems which are exploited to obtain an optimal partition of data. The CMC has already been successfully applied to cluster data in different fields, from medicine to engineering and finance; examples are: the detection of buried land mines using dynamic infrared imaging [7]; the study of human evolution by clustering mitochondrial DNA sequences [8]; the analysis of electroencephalographic signals to recognize Huntington's disease [9]; and the clustering of Dow Jones stock market companies for portfolio optimization strategies [10].

In the present investigation, CMC was used for the first time to analyse protein structures. Recently analysed through different chemometrical approaches aimed at studying the structural differences [3,11-13], the family of MMPs was chosen as a case study. It represented a good benchmark, having a high number of entries in the World Wide Protein Data Bank (wwPDB) [14]. In this regard, it is worth saying that the CMC algorithm is even more accurate when dealing with large number of data. Mostly based on the electrostatic potential similarity, the present study accounted for a number of MMPs higher than previous investigations greatly widening the structural boundaries of the so-called MMP target family landscape [12]. More specifically, previous analyses have been performed on a low number (i.e., 10) of MMPs to evaluate their selectivity on the basis of GRID molecular interaction fields and consensus principal component analyses (CPCA) [3]. Other studies, addressing a higher number of MMPs (i.e., 24, including 15 structures from homology modeling), estimated the similarity within the MMP subsites by taking into account ligand interaction energies [11]. Based again on GRID/CPCA, a further analysis has been reported to evaluate MMP selectivity on a larger number of proteins (i.e., 56 MMPs and 1 TACE) [12]. Finally, some of us carried out the screening of all available MMP structures from the PDB and demonstrated that the analysis of the protein sequences enabled us to reproduce the MMP classification based on the structural domain organization [13]. The present analysis of protein electrostatic potential similarities was shown to be effective in obtaining insight into molecular recognition and substrate specificity. CMC analysis was a successful strategy in landscaping the entire MMP target family as well as in investigating the subsites responsible for molecular selectivity. Despite their diverse fundamentals, the analysis of MMPs via CMC provided satisfying results that generally match, or even outperform, those obtainable by applying standard approach such as the Unweighted Pair Group Method with Arithmetic mean (UPGMA) algorithm. CMC performances were also challenged to analyse MMP primary structures. Finally, CMC made it possible to properly relate molecular shape similarity of the co-crystallised ligands with void volumes available in the X-ray MMP complexes.

## Results and Discussion

### MMP target family landscape

**Figure 1:**
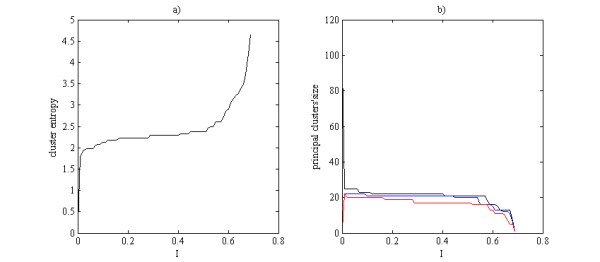
**Choice of the parameter ϑ controlling the resolution at which the data are processed**. (a) Plot of cluster entropy as a function of the mutual information (I). (b) Size of the three largest clusters obtained by the CMC algorithm as a function of the mutual information (I) whose value ranges from 0 to ln2 with a bin-width equal to 0.01.

As a first step, electrostatic potential values calculated on the aligned protein structures were analysed using the CMC algorithm to represent the entire MMP family. In the present analysis, over the course of 10000 iterations, K was heuristically set at 16 while the threshold θ of the mutual information (I) equal to 0.06 was chosen since it intercepted the first and flattest plateau of the cluster entropy [Fig. 1 (a)] yielding the highest member density for the most populated cluster (black line in Fig. 1(b)).

**Figure 2:**
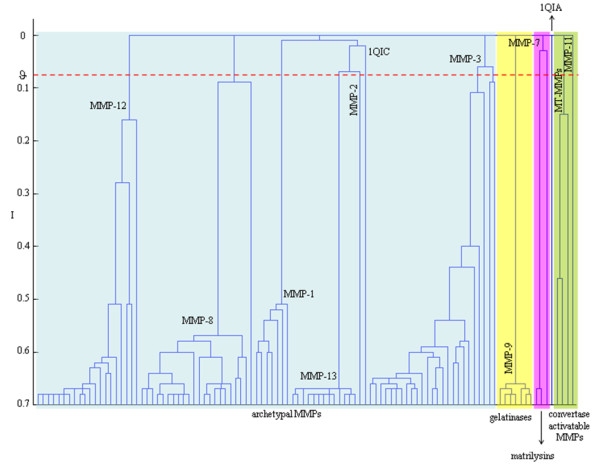
**MMP target family landscape**. Dendrogram obtained from the partition data relative to the electrostatic analysis of the entire protein structures. Different background colour boxes distinguished MMP allocations classified according to the domain organization. Stable clusters at ϑ = 0.06 and K = 16 are labelled with their corresponding MMP class. Singletons are indicated with the PDB codes.

This threshold θ was highlighted through a dashed red line in Fig. 2 and represents the resolution at which the data are analysed or, in other words, indicates the boundary to consider a given structure as a singleton. CMC demonstrated high sensitivity in portraying the MMP target family landscape by properly recognising proteins with similar structural motifs among the different MMP subfamily. When considering domain organization [4], assignment of the MMP structures almost perfectly matched the classification (Fig. 2) with the single exception of a gelatinase (PDB:1QIB) that was misplaced in the group of archetypal MMPs. This misallocation was however due to the inclusion of a short peptide, Ser187-Leu-Gly-Lys-Gly-Val191, instead of the three fibronectin modules enabling specific binding with collagen. This finding emerged also in a previous work concerned with sequence analysis [13]. Interestingly, closer analysis revealed that collagenases as well as MT-MMPs and stromelysins were properly grouped in full agreement with the classification based on MMP subfamilies.

Then, it was observed that MMPs of identical class were aggregated into highly homogenous groups, except for two singletons determined by two stromelysins whose X-ray structures (PDB:1QIA and PDB:1QIC) were missing from a stretch of six residues (i.e., Phe83-Arg-Thr-Phe-Pro-Gly88).

The comparison of CMC and UPGMA (see additional file 1) revealed that congruent results were obtained. However, the dendrogram generated via CMC was indeed more easily interpretable and, to some extent, more informative. Unlike CMC, UPGMA was in fact unable to generate the classification based on the domain organization [4], which is known as the highest level of MMP classification, but also failed to properly cluster MMP-8 and MMP-1 (i.e., 1HFC joined first the unique MMP-2 and then the group formed by MMP-3). Similarly, the analysis carried out by UPGMA confirmed that 1QIA and 1QIC were effectively diverse from the other elements of the same class (i.e., MMP-3) and produced a cladogram (see additional files 1) with the longest branches for these two proteins.

### CMC analyses of MMP binding sites

The second stage of our investigation was focused on MMP active sites and a number of independent CMC analyses were carried out for studying: a) the significant role of the S_1_' pocket in determining enzyme specificity; b) the residues involved in the S_2_-S_2_' stretch embedding the catalytic domain responsible for protein function regulation; c) the region involving S_3_-S_1_-S_3_' subsites constituting a shallow region containing β-strand IV and two slightly variable loops among different MMP isoforms.

#### a) Analysis of the S_1_' subsite

**Figure 3:**
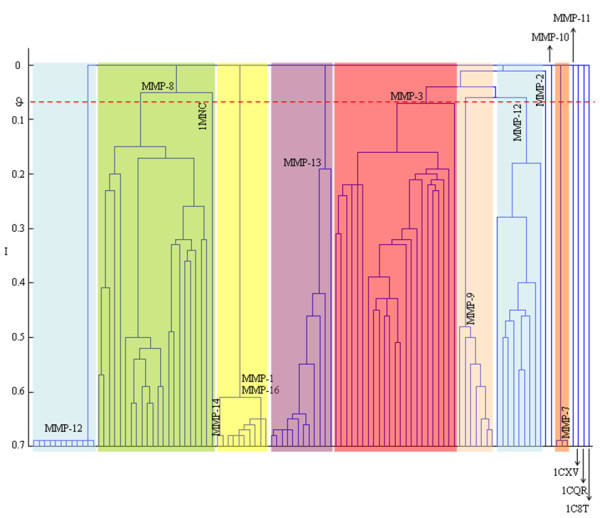
**Analysis of the S_1_' specificity pocket**. Dendrogram obtained from the partition data relative to the electrostatic analysis of the S_1_' subsite. Different background colour boxes were used to distinguish the main stable groups at ϑ = 0.08, by setting K = 10. Singletons are indicated with the PDB codes.

Known as the specificity pocket, the S_1_' subsite is the most relevant cavity within the MMP active site and is characterised by a loop behind such a pocket [13]. CMC analysis focused on 16 residues (from position 217 to position 231 according to MMP-8 numbering) whose consensus sequence PLYHSFTDLTRFRLSQ, obtained through multiple sequence alignment, disclosed rather lower percentages for most of the amino acids [13]. The study of such a variable residue composition is fundamental for interpreting the structural implications responsible for MMP selectivity. In this regard, CMC was successful in properly resolving the different MMP classes through variations of the corresponding key-point residues. As shown in Fig. 3, MMPs of identical class were grouped into a single subgroup with a high level of similarity and, more importantly, were clearly distinguished from all the others. Interestingly, the assignments obtained through the CMC S_1_' focused analysis was, again, better interpretable than those attainable via UPGMA (see additional file 2) which failed in grouping the MMP-3 and two MMP-8 (i.e., 1JH1 and 1MNC that firstly linked the unique MMP-10 and then the MMP-1). However, a closer look at the dendrogram of Fig. 3 revealed that MT-MMPs (i.e., MMP-14 and MMP-16) formed a single group with MMP-1. Such an observation held true also when applying the UPGMA method. Moreover, it was observed that metallo-elastases were split into two different groups containing 12 and 8 members, respectively, exhibiting different side-chain conformations of polar residues Arg249 and Lys 241 (MMP-12 numbering). Similarly, it was observed that the MMP-8 cluster required lower similarity values to incorporate the PDB:1MNC crystal protein with a different side-chain conformation of Glu233 (MMP-8 numbering). In addition, the CMC algorithm designated three structures as singletons. Two of these were stromelysin-1 (i.e., PDB:1C8T, PDB:1CQR), whereas the third was a collagenase-3 (i.e., PDB:1CXV). In particular, the 1C8T structure exhibited a different conformation of the region encompassing Leu229-Thr-Arg-Phe-Arg233 of the loop at the bottom of the S_1_' pocket despite the consistent overlap of protein backbone atoms. Visual inspection of the PDB:1CQR crystal revealed that it was an apo-form and, unlike other stromelysin-1, presented a more restricted and hampered loop. Interestingly, such an observation was also reported in a recent analysis based on the GRID/CPCA approach [12]. In addition, the CMC algorithm recorded that residues Thr247-Gly-Lys-Ser-His251 (MMP-13 numbering) were absent in the PDB:1CXV structure that was thus left as a singletons in the dendrogram.

Finally, a value of global electrostatic similarity [15] for the S_1_' subsite was measured applying the following formula:

(1) $${\text{C}}_{\text{SIM}}=\frac{\sum_{\text{i}=1}^{\text{N}-1}\sum_{\text{j}=\text{i}+1}^{\text{N}}{\text{SI}}_{\text{i,j}}^{}}{\text{N}\left(\text{N}-1\right)/2}$$

where SI is the Hodgkin index of all the protein pairwise (i.e., *i *and *j*) combinations and N is the total number of protein structures. Being SI commutative, C_SIM _accounts for N(N-1)/2 calculations thus avoiding double counts.

**Figure 4:**
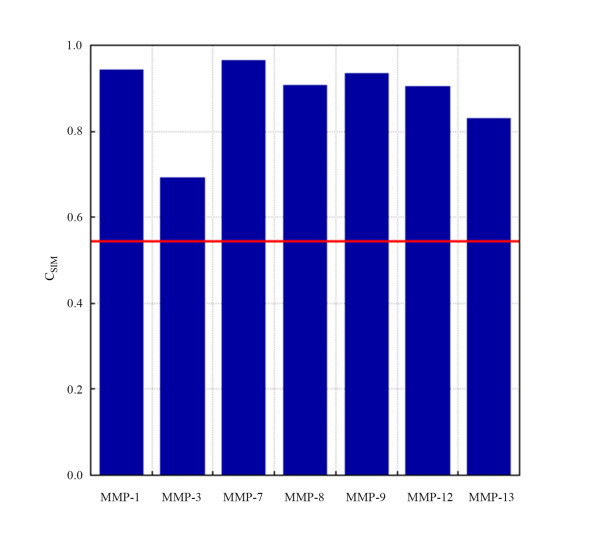
**Representation of global electrostatic similarity at the S_1_' subsites**. The intra-class and inter-class levels are indicated by the blue-coloured bars and solid red line, respectively. Specificity of S1' subsite demonstrated by its higher level of intra-class similarity compared with inter-class similarity.

The analysis involved the MMP classes with N > 1 and resulted in the following decreasing similarity order: MMP-7 (C_SIM _= 0.946, N = 3) > MMP-1 (C_SIM _= 0.933, N = 7) > MMP-9 (C_SIM _= 0.923, N = 7) > MMP-8 (C_SIM _= 0.902, N = 22) > MMP-12 (C_SIM _= 0.899, N = 20) > MMP-13 (C_SIM _= 0.815, N = 13) > MMP-3 (C_SIM _= 0.678, N = 26). The global electrostatic similarity over all the 104 S_1_' subsites was equal to 0.539. These results underlined the relevance of the S_1_' subsite for molecular selectivity, as higher similarity values were observed within each class (intra-class similarity) while the similarity among all the classes was significantly lower (inter-class similarity) [Fig. 4].

#### b) Analysis of the S_2_-S_2_' subsites

The S_2_-S_2_' protein regions represented the catalytic domain and displayed a more pronounced similarity (C_SIM _= 0.949, N = 104) compared to the S_1_' subsite. As expected, this remarkable electrostatic similarity value was directly related to the consistent percentages of residue consensus.

Although the detection of electrostatic differences proved even more difficult, CMC was able to perceive the variation of electrostatic potential of diverse polar residues. Encompassing residues from position 197 to position 207 (MMP-8 numbering), the stretch under investigation is the well-known sequence motif, HExGHxxGxxH, which is common to all MMPs. As expected, the presence of charged residues among variable residues was immediately detected by the CMC algorithm. For instance, the presence of negatively charged residues (i.e., Glu, Asp) at position 206 implied a consistent variation of the electrostatic potential that was immediately perceived by the CMC algorithm, which resulted in clearly distinct groups including gelatinases, MT-MMPs and MMP-13. In addition, the CMC was able to detect the occurrence of different side-chain conformations and even punctual residue mutations. For instance, gelatinases were split into two groups. The first collected MMP-9 structures incorporating the E402Q mutation (MMP-9 numbering) while the second group contained the only wild MMP-9 structure (PDB:1GKC) and the only X-ray MMP-2 (PDB:1QIB) structure. Moreover, MMP-14 included a member (PDB:456C) of the MMP-13 class. Such a crystal structure differed from other MMP-13 proteins since it lacked residues 104 to 109 (MMP-13 numbering), whose remaining available space was occupied by the Asp421 side-chain exhibiting a diverse conformation. Furthermore, the MMP-12 group did not include two structures (PDB:2W0D and PDB:1JK3) for the occurrence of the E219A mutation (MMP-12 numbering). Interestingly, the UPGMA method afforded comparable results. In this regard, the obtained cladogram (see additional file 3) associated such elements with clearly distinguishable longer branches emerging from a fairly flat tree-like plot.

#### c) Analysis of the S_3_-S_1_-S_3_' subsites

**Figure 5:**
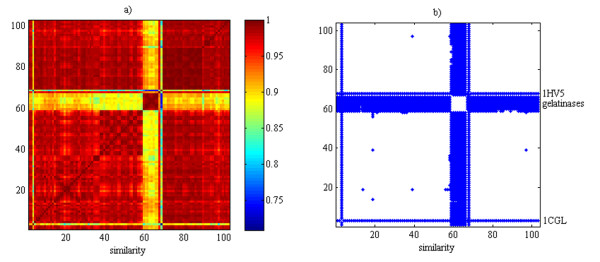
**High electrostatic similarity of S_3_-S_1_-S_3_' stretches apart from gelatinases subfamily**. (a) Heat map relative to the pairwise electrostatic distances for S_3_-S_1_-S_3_' analysis. Colours ranging from red to blue indicate shifts from high to low similarity values. (b) Spy plot of the similarity values lower than 0.93; those greater than the threshold were dropped to zero.

The residue stretches comprising the S_3_-S_1_-S_3_' subsites disclosed a higher electrostatic similarity (C_SIM _= 0.964) compared to the S_1_' subsite. In this regard, the heat map in Fig. 5 (a) furnished an immediate idea of the overwhelming dominance of red colours indicating high electrostatic similarity at the S_3_-S_1_-S_3_' subsites. However, the presence of somewhat limited green-like coloured regions was observed for similarity values lower than 0.93. By zeroing values greater than 0.93, a spy plot [Fig. 5 (b)] was obtained to conveniently illustrate the high internal similarity of gelatinases as well as their remarkable differences compared with other MMPs. Nevertheless, it has to be again remarked that such results are in full agreement with those obtained when applying the UPGMA method. In this regard, by inspecting the cladogram obtained via UPGMA (see additional file 4), the interested reader can appreciate that the high inter-class similarity is proved by the occurrence of short links among all MMP structures, with the exception of the gelatinases, as discussed above.

**Figure 6:**
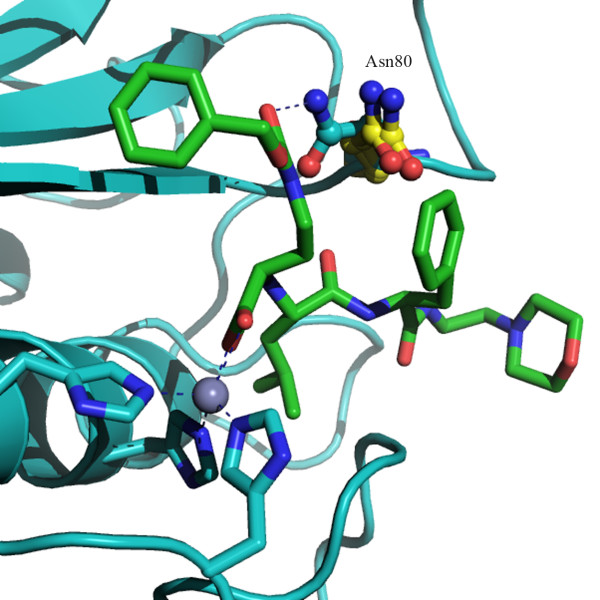
**Induced fit phenomena differentiates structures belonging to the same MMP class**. Elucidation of differences between PDB:1CGL with PDB:1CGE and PDB:1CGF when comparing S_3_-S_1_-S_3_' regions. The Asn80 residue is coloured yellow for the apo-proteins (PDB:1CGF and PDB:1CGE) and cyan for the complex (PDB:1CGL) co-crystallised with a peptide inhibitor rendered as green sticks. The coordination of zinc with the three catalytic histidines and carboxylic group of the ligand is highlighted.

A close assessment of the spy plot disclosed a number of other valuable clues. For instance, the only X-ray structure of MMP-11 (i.e., PDB:1HV5) was clearly distinguished from all the others; such a result was in agreement with previous investigation [13] based on rmsd analyses. Similarly, the PDB:1CGL crystal belonging to MMP-1 displayed a pronounced dissimilarity compared to the other members of the same class for the likely occurrence of induced fit phenomena. As shown in Fig. 6, the Asn80 residue was shifted away from its location observed in apo-forms of the same structure (i.e., PDB:1CGE and PDB:1CGF) for the likely occurrence of a hydrogen bond interaction with the carbonyl group of co-crystallised peptide inhibitor. In this regard, it is worth saying that similar considerations can be drawn by inspecting the cladogram generated via UPGMA (see additional file 4). The CMC and UPGMA methods differed remarkable in assigning the PDB:1YOU structure which is completely isolated in the cladogram resulting from UPGMA and is able to join only the PDB:1HV5 through a very long link. In this regard, the CMC assignment seems to be more interpretable since the PDB:1YOU, which belongs to MMP-13, is inserted in the cluster of the MMP-13 and is directly joined to two of them (i.e., PDB:2PJT and PDB:1ZTQ). Interestingly, the inspection of the PDB:1YOU structure revealed that its Tyr195 residue was differently oriented with respect to the other MMP-13 and was instead closer to the PDB:2PJT and PDB:1ZTQ.

### CMC analysis of MMP primary structures

**Figure 7:**
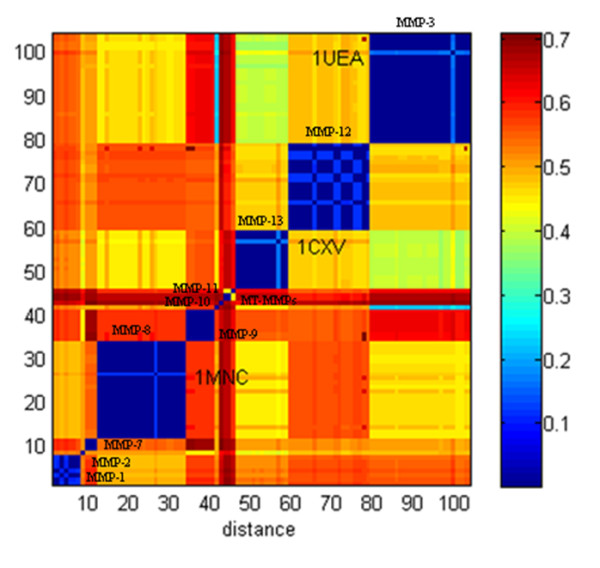
**CMC analysis of MMP sequences**. Sequence-based clustering of the MMP family. The level of sequence distance is indicated by the colour shift from blue to red. Proteins classes were labelled, PDB codes were referred to those sequences assigned as singletons during CMC analysis.

CMC was also challenged to evaluate its ability to group proteins on the basis of their sequence similarity. Pairwise sequence distances were represented through a heat map (Fig. 7). As can be seen, high similarity was generally measured in each class. Nevertheless, some sequences (i.e., PDB:1UEA, PDB:1MNC and PDB:1CXV) did not meet this trend and thus the CMC algorithm effectively detected them as singletons despite their proximity to other proteins of the same type. Moreover, it was observed that MMP-1 and MMP-12 were both split into two different subclusters. This was in part due to the occurrence of punctual mutations (e.g., E219A, F171D) or missing residues so that sequences belonging to two separate clusters were similar at 99.4%.

### Ligand analysis via molecular shape similarity

The CMC algorithm was finally used to analyse the molecular shapes of the 84 co-crystallised ligand molecules extracted from the pool of the 104 examined X-ray MMP structures. The present analysis was aimed at evaluating binding specificity towards MMPs on the basis of the complementarity between void volumes within the MMP binding sites and the molecular shapes of the co-crystallised inhibitors. Specifically, CMC made it possible to relate molecular shape similarity with even subtle diversity of MMP physicochemical environments based on the fundamental assumption that two ligand molecules would have the same shape if their volumes matched exactly.

**Figure 8:**
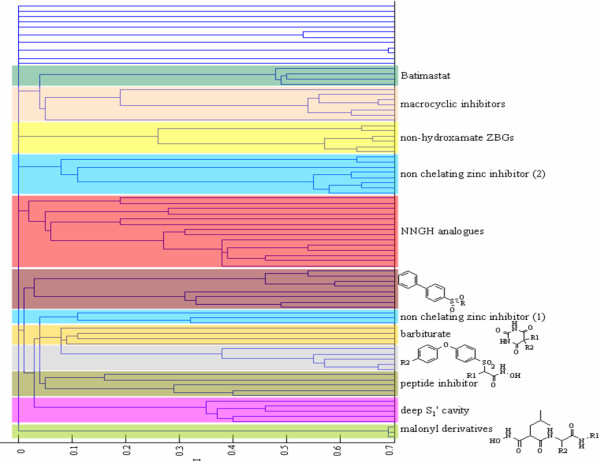
**Analysis of co-crystallised ligands via molecular shape similarity**. Dendrogram obtained from the partition data relative to shape similarity analysis of the 84 co-crystallised ligand molecules. Different colours were used to distinguish the main stable groups at ϑ = 0.08, by setting K = 2.

The CMC algorithm allowed us to obtain a dendrogram encoding various informative levels (Fig. 8). Firstly, the co-crystallised inhibitors were assigned according to the their chemical structure, since the observed clusters contain structurally similar inhibitors (e.g., malonyl derivatives, barbiturates, 2-benzenesulfonylamino-N-hydroxy-acetamides, macrocyclic derivatives and so on). Secondly, the CMC was able to discern zinc chelating inhibitors from non-zinc chelating inhibitors regardless of MMP class. The vast majority of reported MMP inhibitors are zinc chelating compounds, whose X-ray structures reveal that inhibitor binding interactions typically include coordination of the catalytic zinc ion and occupancy of the S_1_' pocket with a hydrophobic group [16]. However, these are broad spectrum inhibitors lacking selectivity. In the development of new therapeutic agents targeting MMPs, there remains considerable room for new structural classes not coordinating the active site zinc atom [17]. In this regard, exploiting the structural differences between the MMP subtypes, currently an important issue in this area, should lead to more specific inhibitors, intended to avoid the toxic side effects supposedly linked to broad spectrum inhibitors [18].

Thirdly, low molecular selectivity was immediately inferred by observing a high concentration of replicates (i.e., batimastat and NNGH analogues) within the same cluster, although these co-crystallised with different MMPs. Finally, the analysis of ligand molecular shapes via CMC algorithm made it possible to gain insight into selectivity beyond the backbone on the basis of the size (i.e., shallow or deep) of the S_1_' pocket [19]. Inhibitors with bulkier moieties (e.g., 4-(4-phenyl-piperidin-1-yl)-benzenesulfonylamino and 4'-[(benzofuran-2-carbonyl)-amino]-biphenyl-4-sulfonylamino substituents) were effectively comprised in the same cluster intercepting deep pocket MMP complexes (i.e., PDB:1B8Y, PDB:1CIZ and PDB:1CAQ as MMP-3, PDB:1ZTQ and PDB:1ROS as MMP-12).

## Conclusions

The main objective of the present investigation was to apply the chaotic map algorithm to clustering MMP structures. Based on electrostatic potential values, CMC analyses afforded a comprehensive representation of the intimate relationships existing among MMPs, showing that structural differences between proteins reflect genuine variation of their electrostatic potentials. In particular, CMC analysis of entire MMP structures was successful in accurately reproducing the canonical classification of MMPs normally based on domain organization. Such a result was not attained when the analysis was repeated by using the UPGMA approach. In addition, CMC demonstrated high sensitivity in discerning even smaller protein stretches, and defining relevant areas in proximity to the binding site. More importantly, CMC was able to properly detect the variance of electrostatic potential occurring for even punctual mutations of ionisable residues. Furthermore, CMC demonstrated an outstanding aptitude for capturing local distortions of the electrostatic potential probably related to physical incorporation of small ligands inducing smaller structural protein rearrangements. Interestingly, CMC represented a valid strategy even for standard analyses as those involving only MMP sequences. Similarly, CMC was successful in correctly relating the molecular shapes of ligand molecules to the void volumes available within the MMP binding sites.

In this view, CMC could represent a valuable alternative approach or a complement to other clustering methods assessing the structural similarity within protein families. CMC demonstrated performances comparable to those of the UPGMA, with the former leading however to more easily interpretable results. Incidentally, it should be said that CMC is tailored to deal with large amounts, and could also have potential in the database mining.

## Methods

### Data set

The protein data set is made of 104 MMP 3D structures available from the wwPDB [14] and listed in Table 1. The herein presented collection of MMPs encompassed all the structures previously published [3,10-13].' In cases where the asymmetric unit contained more than one protein occurrence, the first structural copy of the biological unit was considered. Structures resulting from NMR analysis were intentionally not taken into account, in order to minimize the risk of introducing noise in the data set, and to avoid bias in selecting just one Cartesian snapshot among those collected in solution.

**Table 1:** List of X-ray solved MMP structures retrieved from the wwPDB.

	sub-families	classes	PDB codes
archetypal MMPs		MMP-1	1CGE, 966C, 1HFC, 2TCL, 1CGL, 1CGF, 2J0T
	Collagenasi	MMP-8	1MNC, 1ZS0, 1ZP5, 1MMB, 1JAO, 1JAP, 1JAQ, 1JJ9, 1I76, 1I73, 1ZVX, 1BZS, 1KBC, 1JAN, 1A86, 1A85, 1JH1, 3DNG, 3DPE, 3DPF, 2OY2, 2OY4
		MMP-13	1XUC, 1XUR, 1XUD, 1YOU, 830C, 456C, 1ZTQ, 2D1N, 1CXV, 2PJT, 2OW9, 2E2D, 2OZR
	metallo-elastase	MMP-12	1Y93, 1RMZ, 1OS9, 1OS2, 1UTZ, 1UTT, 1JIZ, 1ROS, 1JK3, 3F15, 3F16, 3F17, 3F18, 3F19, 3F1A, 2W0D, 2HU6, 2OXU, 2OXW, 2OXZ
	Stromelysin	MMP-3	1B8Y, 1CIZ, 1CAQ, 1G4K, 2USN, 1USN, 1SLM, 1UEA, 1HFS, 1QIC, 1C3I, 1CQR, 1BQO, 1BIW, 1SLN, 1HY7, 1G05, 1G49, 1D5J, 1D8F, 1D7X, 1D8M, 2D1O, 1B3D, 1QIA, 1C8T
		MMP-10	1Q3A
matrilysin	Matrilysin	MMP-7	1MMP, 1MMQ, 1MMR
gelatinases	Gelatinase	MMP-9	1GKC, 1GKD, 2OVX, 2OVZ, 2OW0, 2OW1, 2OW2
		MMP-2	1QIB
convertase-activatable MMPs	MT-MMPs	MMP-14	1BUV, 1BQQ
		MMP-16	1RM8
	stromelysin-3	MMP-11	1HV5

After removing water molecules and co-crystallised inhibitors, MMPs were aligned onto Cartesian coordinates of C-alpha atoms and the three catalytic histidine side chains of 1ZS0, selected as template.

In addition, the ligand data set comprised a number of 84 co-crystallised ligands extracted from the pool of X-ray MMP structures.

### Protein electrostatic potential similarity

According to a recent work [19], each protein structure was subjected to electrostatic potential calculation by using Adaptive Poisson-Boltzmann Solver (APBS) program [20]. A grid of dimensions 65 × 65 × 65 Å^3 ^was used, together with a 1.5 Å grid spacing, for the computation of the electrostatic potential via a finite difference solution of the linearised Poisson-Boltzmann equation. The grid was centred on the global centre of mass of the superimposed structures. The dielectric constants of the solvent and the protein were set to 78 and 1, respectively. Charges were assigned by using AMBER99 force field and hydrogen bonding network optimization was not set to keep unchanged the protonation state of all polar residues [21].

Using default parameters, Protein Interaction Property Similarity Analysis (PIPSA) software [22] was run to obtain distance matrix. Electrostatic potentials (M) were computed at points (x, y, z) on a three-dimensional grid surrounding the entire protein structures. On the basis of the electrostatic potential values, Similarity Indices (SI) were then computed for grid points within the intersection of a specific region, defined as "skin", surrounding each MMP structure at a distance of 3Å from the van der Waals surface and having a thickness of 4 Å. As documented [22], the use of the skin region enabled to better account for the protein similarity shape.

For all the n(n - 1)/2 possible pairwise comparisons, electrostatic similarity was computed by applying the Hodgkin index [23] as follows:

(2) $${\text{SI}}_{\text{i,j}}^{}=\frac{2({\text{M}}_{\text{i}},{\text{M}}_{\text{j}})}{{\text{M}}_{\text{i}}^{2}+{\text{M}}_{\text{j}}^{2}}$$

being (M_i_, M_j_), ${\text{M}}_{\text{i}}^{2}$, and ${\text{M}}_{\text{j}}^{2}$ scalar products.

It is easy to demonstrate that when two potentials are identical then SI_i,j _= 1, when they are uncorrelated SI_i,j _= 0 and when they are anti-correlated SI_i,j _= - 1.

Similarity matrix was, therefore, converted into the correspondent distance matrix by applying the following widely accepted equation:

(3) $${\text{D}}_{\text{i,j}}=\sqrt{2(1-{\text{SI}}_{\text{i,j}})}$$

where, for each given pair of proteins, SI_i,j _and D_i,j _represent the similarity and distance values, respectively. The latter were effectively used as input for the CMC algorithm.

### Sequence similarity

The Smith-Waterman algorithm [24] was used for aligning primary structures by selecting the PAM250 scoring matrix and setting gap-open, gap-extend, and scale value at 10.0, 0.5 and 3.0, respectively. As already done for electrostatic potential similarity values, the obtained matrix was converted into the corresponding distance matrix through the equation 3.

### Ligand molecular shape similarity

The molecular shape and the pairwise similarity analysis of the ligand data set was operated through the program ROCS (standing for Rapid Overlay of Chemical Structures, from OpenEye Scientific Software) [25], disabling solid-body optimization process to maintain unchanged the protein-ligand positions. The Tanimoto indexes calculated for all the n(n - 1)/2 pairwise MMP ligand combinations were, thus, converted into distance values by applying equation 3. The obtained data were stored into a square matrix for running CMC algorithm.

### Chaotic map clustering algorithm

Written in MATLAB metalanguage (The MathWorks, Inc.) [26], the CMC algorithm was originally introduced as a central algorithm, where the elements to cluster are embedded in a *D*-dimensional feature space. In such a picture, the data-points are viewed as sites of a grid, hosting a chaotic map dynamics. Depending on the analysis carried out, the entire protein structures, the protein subsites, the sequences or the ligand structures are thus used as input data-points which are distributed in a vectorial space so that a map variable x_i _∈ [-1,1], i = 1...N can be assigned to each structure. Initially, the assignment is purely random. The entire system will then evolve on the basis of the short range interactions between neighbouring maps. In this respect, the diverse distance D_ij _associated to the different analyses is used to measure the corresponding data coupling J_ij _= exp [-(D_ij_)^2^/2a^2^], where *α *is the local length scale, whose value is the average distance of the K-nearest neighbours. Being J_ij _an exponential decreasing function of the site distance D_ij_, a high value of the distance stands for a low tendency of coupling. In the present study, a pairwise version of the algorithm was implemented by simply adopting the distance matrix described above, in the equation of the couplings J_ij_. The parameter K is set at a value such that its change does not affect substantially the clustering results. This value is independent of the size of the dataset, rather it depends on the particular distribution of the data at hand.

The dynamics of the system, leading to the formation of synchronized maps in correspondence of points close in the feature space, is given by

(4) $${\text{x}}_{\text{i}}(t+1)=\frac{1}{{\text{C}}_{\text{i}}}\sum_{j\neq i}{\text{J}}_{\text{ij}}f({\text{x}}_{\text{j}}(\text{t}))$$

where C_i _= ∑_j≠i _J_ij _is a normalization factor and *f*(x) = 1 - *α*x^2 ^is the logistic map with *α *= 2. Starting from a random configuration {x_i_}, the last equation is iterated until the system attains its stationary regime when clusters of synchronized maps emerge out. Once the algorithm completed a given predetermined number of iterations, a similarity index for clustering the data is obtained through the value of the mutual information I_ij _between maps. The mutual information can be calculated after defining, for each evolving map x_i_(*t*), a bit sequence S_i _≡ {0,1} such that S_i _= 1 if x_i_(*t*) ≥ 0, and 0 otherwise. The dichotomic values occurring into the bit sequence allow the evaluation of the Shannon entropy H_i_, for each map x_i_(*t*), and the joint entropy H_ij_, for pairs of maps *i *and *j *as follows:

(5) $$\begin{array}{l} {\text{H}}_{\text{i}}=-\sum_{\text{Si}=0,1}\text{P}({\text{S}}_{\text{i}})\ln\text{P}({\text{S}}_{\text{i}})\\ {\text{H}}_{\text{ij}}=-\sum_{\text{Si}=0,1}\sum_{\text{Sj}=0,1}\text{P}({\text{S}}_{\text{i}},{\text{S}}_{\text{j}})\ln\text{P}({\text{S}}_{\text{i}},{\text{S}}_{\text{j}}) \end{array}$$

where P(S_i_) is the probability of the state S_i _≡ {0,1} along the bit sequence *i*, computed as the fraction of occurrence; likewise, P(S_i_,S_j_) is the probability that the pair {S_i_,S_j_} ≡ {(0,0),(0,1),(1,0),(1,1)} occurs at the same step, along two sequences *i *and *j*. The mutual information is then given as follows:

(6) $${\text{I}}_{\text{ij}}={\text{H}}_{\text{i}}+{\text{H}}_{\text{j}}-{\text{H}}_{\text{ij}}$$

The mutual information is a measure of the correlations between maps, ranging from I_ij _= 0 for independently evolving maps to I_ij _= ln 2 for exactly synchronized maps. In the stationary regime, a link is set between all pairs of data points whose associated maps satisfy the condition I_ij _> ϑ, where ϑ is a threshold. The clusters are identified as the linked components of the graph, thus, obtained. The parameter controls the resolution at which the data are processed: sweeping its value throughout the range [0, ln2] a hierarchical solution is obtained. Each clustering level corresponds to a partition of the data; the optimal solution among the whole hierarchy yielded by the algorithm is selected as the most stable one, and the corresponding value of ϑ is set at the flattest plateau in the plot of the cluster entropy, defined as follows:

(7) $$\text{S}\left(\vartheta \right)=-\sum_{\text{i}}\frac{{\text{N}}_{\text{i}}\left(\vartheta \right)}{\text{N}}\ln\frac{{\text{N}}_{\text{i}}\left(\vartheta \right)}{\text{N}}$$

where N_i_(ϑ) is the number of elements in the *i*-th cluster at threshold ϑ and N is the total number of elements.

### Unweighted Pair Group Method with Arithmetic mean

To compare CMC performance with those achievable through others clustering methods, UPGMA was used as it represents a standard clustering bioinformatics approach. Moreover, it was adopted to generate Phylip representations [27] from a distance matrix within the PIPSA package [22]. The UPGMA constructs a rooted tree by using the average-linkage as metric of clustering. At each step, the nearest two clusters are combined into a higher-level cluster. The distance between any two clusters A and B is taken to be the average of all distances between pairs of objects *x *in A and *y *in B, that is, the mean distance between elements of each cluster.

## Authors' contributions

IG carried out all the calculations and modeling; IG, ON, AC and RB assessed the accuracy of modeled structures; FDC, GG and RB designed the initial CMC algorithm while IG and ON developed the pairwise variant; ON and AC participated in the design and coordination of the study; ON supervised the analysis of results and wrote the manuscript. All the authors have read and approved the final version of the manuscript.

## Supplementary Material

Additional file 1 **UPGMA tree representation of the MMP target family landscape**. Cladogram obtained from the partition data relative to the analysis of electrostatic potential similarity applied to the entire protein structures. Click here for file

Additional file 2 **UPGMA tree representation of the S_1_' specificity pocket**. Cladogram obtained from the partition data relative to the electrostatic analysis of S_1_' subsite. Click here for file

Additional file 3 **UPGMA tree representation of the S_2_-S_2_' subsites**. Cladogram obtained from the partition data relative to the electrostatic analysis of S_2_-S_2_' subsites. Click here for file

Additional file 4 **UPGMA tree representation of the S_3_-S_1_-S_3_' subsites**. Cladogram obtained from the partition data relative to the electrostatic analysis of S_3_-S_1_-S_3_' subsites. Click here for file
